# Early Gestational Weight Gain Rate and Adverse Pregnancy Outcomes in Korean Women

**DOI:** 10.1371/journal.pone.0140376

**Published:** 2015-10-14

**Authors:** Eun-Hee Cho, Junguk Hur, Kyung-Ju Lee

**Affiliations:** 1 Department of Internal Medicine, Kangwon National University, Kangwon-do, Korea; 2 Department of Biomedical Sciences, University of North Dakota, School of Medicine and Health Sciences, Grand Forks, North Dakota, United States of America; 3 Department of Obstetrics and Gynecology, Kangnam CHA Medical Center, CHA University, Seoul, Korea; 4 Integrative Medicine Center, Korea University Medical College, Seoul, Korea; CHA University, REPUBLIC OF KOREA

## Abstract

During pregnancy, many women gain excessive weight, which is related to adverse maternal and neonatal outcomes. In this study, we evaluated whether rate of gestational weight gain (RGWG) in early, mid, and late pregnancy is strongly associated with adverse pregnancy outcomes. A retrospective chart review of 2,789 pregnant Korean women was performed. Weights were recorded at the first clinic visit, during the screening test for fetal anomaly, and during the 50g oral glucose challenge test and delivery, to represent early, mid, and late pregnancy, respectively. A multivariate logistic regression analysis was performed to examine the relationship between RGWG and adverse pregnancy outcomes. At early pregnancy, the RGWG was significantly associated with high risk of developing gestational diabetes mellitus (GDM), pregnancy-induced hypertension (PIH), large for gestational age (LGA) infants, macrosomia, and primary cesarean section (P-CS). The RGWG of mid pregnancy was not significantly associated with any adverse pregnancy outcomes. The RGWG at late pregnancy was significantly associated with a lower risk of developing GDM, preterm birth and P-CS, but with a higher risk of developing LGA infants and macrosomia. When the subjects were divided into three groups (Underweight, Normal, and Obese), based on pre-pregnancy body mass index (BMI), the relationship between early RGWG and adverse pregnancy outcomes was significantly different across the three BMI groups. At early pregnancy, RGWG was not significantly associated to adverse pregnancy outcomes for subjects in the Underweight group. In the Normal group, however, early RGWG was significantly associated with GDM, PIH, LGA infants, macrosomia, P-CS, and small for gestational weight (SGA) infants, whereas early RGWG was significantly associated with only a high risk of PIH in the Obese group. The results of our study suggest that early RGWG is significantly associated with various adverse pregnancy outcomes and that proper preemptive management of early weight gain, particularly in pregnant women with a normal or obese pre-pregnancy BMI, is necessary to reduce the risk of developing adverse pregnancy outcomes.

## Introduction

During pregnancy, many women gain excessive weight [1], and gestational weight gain (GWG) is related to adverse maternal and neonatal outcomes [2–5]. Strong relationships between excessive GWG and increased birth weight and large-for-gestational-age (LGA) infants have been reported [4]. Obese women with low gestational weight gain had a decreased risk for preeclampsia, cesarean section, and LGA infants, but women with more than 16 kg GWG showed an increased risk for cesarean section in all maternal body mass index (BMI) classes [6]. A recent study showed that mid-gestational weight gain was a strong predictor for birth weight and neonatal subcutaneous fat [7]. Another study demonstrated that the GWG was significantly associated with obesity for the offspring at the age of eight years [8]. However, there are few studies of the relationship between early GWG and gestational diabetes mellitus (GDM) [9–11] and GWG prior to glycemic screening and maternal hyperglycemia [10, 11]. Our objective was to examine if the rate of GWG (RGWG) in different pregnancy stages (early, mid, and late) is strongly associated with adverse pregnancy outcomes.

## Materials and Methods

This study used data from pregnant women who delivered between July 1, 2007 and December 31, 2009 at CHA Kangnam Medical Center (Seoul, Korea). Subjects with twin pregnancy, fetal anomaly, hypertensive disorder before pregnancy, preexisting diabetes, and missing pre-pregnancy or weight at delivery were excluded. The total number of subjects included for further analyses was 2,789. Gestational age was estimated based on the reported last menstrual period and adjusted with fetal crown-rump length (CRL) measured in early pregnancy. Height was measured at the first clinic visit. The weights used in the present study included self-reported pre-pregnancy weight and measured weights during the clinic visits at the time of the screening test for fetal anomaly, 50 gram oral glucose challenge tests (OGCTs), and delivery. Blood pressure was measured at each clinic visit. Typically at CHA hospital, stable blood pressure readings, taken after minimum ten minute resting, are obtained from patient's upper left arm using an appropriately-sized cuff. The complete anonymized data are available in S1 File.

**Fig 1:**
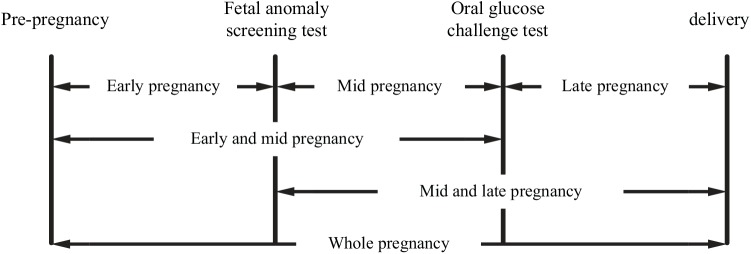
Times of weight measurement and pregnancy term definition. The rate of gestational weight gain (lb/week) was calculated for terms defined as followings: early pregnancy (from pre-pregnancy to fetal anomaly screening test), mid pregnancy (from fetal anomaly screening test to oral glucose tolerance test), late pregnancy (from oral glucose tolerance test to delivery), “early and mid pregnancy” (from pre-pregnancy to oral glucose tolerance test), “mid and late pregnancy” (from fetal anomaly screening test to delivery), and whole.

Instead of using the “typical” three trimesters, we defined three gestational age terms according to routine scheduled visits for pregnant women: early pregnancy (from pre-pregnancy to the screening test for fetal anomaly), mid pregnancy (from the screening test for fetal anomaly to the 50g OGCT), and late pregnancy (from the 50g OGCT to delivery). Rate of gestational weight gate (RGWG; lb/week) was calculated for the following periods (Fig 1): early pregnancy, mid pregnancy, late pregnancy, early and mid pregnancy, mid and late pregnancy, and whole gestation.

### Adverse pregnancy outcomes

Adverse pregnancy outcomes included the following: (1) pre-term birth (delivery at less than 37 weeks’ gestation); (2) GDM (two or more positive results in 3-hour 100g oral glucose tolerance test (OGTT); fasting ≥ 95 mg/dl, 1 hour ≥ 180 mg/dl, 2 hour ≥ 155 mg/dl, and 3 hour ≥ 140 mg/dl); (3) macrosomia (birth weight of 4,000g or greater); (4) large or small for gestational age (LGA or SGA; birth weight > 90 or < 10 percentiles, respectively, defined in Williams *et al*.’s fetal growth table [12]); (5) primary cesarean section (P-CS; due to failure to progress, mal-presentation of fetus, or past history of uterus operation, but excluding repetitive CSs); (6) low 1-min activity, pulse, grimace, appearance, respiration (APGAR) scores less than 5; and (7) pregnancy-induced hypertension (PIH; systolic blood pressure > 140 mmHg or diastolic blood pressure > 90 mmHg after 20 weeks’ gestation).

### Statistical analysis

Data were demonstrated using descriptive statistics as mean ± standard deviation unless otherwise stated. The effects of RGWG during pregnancy on the incidences of adverse outcomes were examined using multinomial logistic regression with age and pre-pregnancy BMI as covariates. A *P*-value < 0.05 was considered statistically significant. For statistical analyses, the R statistical analysis software version 3.2.1 (http://cran.r-project.org/) was used.

### Ethics statement

The Institutional Review Board (IRB) of CHA Kangnam Medical Center (IRB No. KNC 10–025) approved the protocol of this study. Informed consent was not obtained from patient subjects, as patient records/information was anonymized and de-identified prior to analysis.

## Results

The medical records were collected initially for 3,980 pregnancies. A total of 2,789 pregnancies during the study period met our inclusion criteria. Table 1 summarizes the clinical characteristics of the study subjects. The mean age was 33.4 ± 3.8 years old and the mean pre-pregnancy BMI was 21.1 ± 3.0 kg/m^2^. Maternal weights were obtained at early (16.38 ± 0.75 weeks), mid (26.61 ± 1.25 weeks), and late pregnancy (39.05 ± 1.74 weeks) (Fig 1). The RGWGs were 0.28 ± 0.40, 1.15 ± 0.53, and 0.89 ± 0.53 lb/week in early, mid, and late pregnancies, respectively (Table 2).

**Table 1:** Clinical characteristics of study subjects.

Characteristic	Mean ± SD or number
Number of subjects	2,789
Maternal age (years)	33.4 ± 3.8
Parity	
0	1,623 (58.2%)
≥ 1	1,164 (41.8%)
History of hypertension	14 (0.5%)
Family history of DM	31 (1.1%)
Family history of hypertension	530 (19.0%)
Pre-pregnancy BMI (kg/m^2^)	21.1 ± 3.0
Gestational age at (weeks)	
Fetal anomaly screening test	16.4 ± 0.8
50 gram OGCT	26.6 ± 1.2
Delivery	39.0 ± 1.7
Birth weight (g)	3,257.7 ± 466.9

**Table 2:** Average rate of gestational weight gain.

Pregnancy Term	Rate of gestational weight gain (mean ± SD (lb/week))
Early pregnancy	0.28 ± 0.40
Mid pregnancy	1.15 ± 0.53
Late pregnancy	0.89 ± 0.53
Early and mid pregnancy	0.61 ± 0.30
Mid and late pregnancy	1.01 ± 0.35
Whole pregnancy	0.70 ± 0.26

Table 3 lists the odds ratios (ORs) of developing adverse outcomes for each RGWG after adjusting for age and pre-pregnancy BMI. At early pregnancy, the RGWG was strongly associated with a high risk of GDM (OR = 1.77), PIH (OR = 2.80), LGA infants (OR = 1.77), macrosomia (OR = 1.93), and P-CS (OR = 1.65). However, it was not significantly associated with preterm delivery, 1-min APGAR scores, and SGA infants. The RGWG up to mid pregnancy was also strongly highly associated with these adverse outcomes, as well as a lower risk of developing SGA (OR = 0.36). Interestingly, the RGWG at mid pregnancy alone was not significantly associated with any adverse outcomes.

The RGWG at late pregnancy was significantly associated with lower risk of developing GDM (OR = 0.29), pre-term birth (OR = 0.53), and P-CS (OR = 0.74), as well as a higher risk of developing LGA infants (OR = 1.53) and macrosomia (OR = 1.49). Interestingly, the RGWG at late pregnancy showed an opposite pattern of developing GDM (OR = 0.29) and P-CS (OR = 0.74) compared to those from early pregnancy. The RGWG throughout pregnancy was significantly associated with lower risk of developing GDM (OR = 0.54), preterm delivery (OR = 0.39), and SGA infants (OR = 0.15), but with a higher risk of P-CS (OR = 1.54), LGA infants (OR = 5.88), macrosomia (OR = 5.85), and PIH (OR = 3.93).

**Table 3:** Risk analysis of developing adverse pregnancy outcomes.

Adverse pregnancy outcome	GDM	PIH	Preterm	Macrosomia	LGA	SGA	P-CS	Low APGAR
	n = 260 (9.3%)	n = 73 (2.6%)	n = 150 (5.4%)	n = 110 (3.9%)	n = 120 (4.3%)	n = 152 (5.5%)	n = 1423 (51.0%)	n = 107 (3.8%)
**Early pregnancy**	1.77 (1.23–2.55) §	2.80 (1.54–5.09) §	0.92 (0.55–1.58)	1.93 (1.08–3.46) §	1.77 (1.04–3.06) §	0.62 (0.36–1.09)	1.65 (1.27–2.17) §	1.13 (0.57–2.32)
**Mid pregnancy**	0.93 (0.71–1.23)	1.21 (0.77–1.89)	1.29 (0.83–1.97)	1.51 (0.96–2.33)	1.48 (0.96–2.24)	0.72 (0.48–1.12)	1.16 (0.94–1.43)	1.16 (0.65–2.00)
**Late pregnancy **	0.29 (0.21–0.40) §	1.16 (0.76–1.68)	0.53 (0.37–0.79) §	1.49 (1.04–2.12) §	1.53 (1.08–2.15) §	0.76 (0.50–1.17)	0.74 (0.60–0.90) §	0.67 (0.42–1.09)
**Early and mid pregnancy**	2.22 (1.43–3.47) §	4.46 (2.14–9.16) §	0.91 (0.45–1.79)	3.09 (1.49–6.28) §	2.65 (1.31–5.26) §	0.36 (0.17–0.77) §	3.16 (2.19–4.58) §	1.33 (0.58–2.95)
**Mid and late pregnancy**	0.22 (0.14–0.33) §	1.44 (0.73–2.81)	1.24 (0.65–2.32)	2.91 (1.54–5.43) §	2.94 (1.62–5.32) §	0.50 (0.26–0.97) §	0.81 (0.59–1.09)	0.91 (0.40–2.07)
**Whole pregnancy**	0.54 (0.32–0.89) §	3.93 (1.66–9.13) §	0.39 (0.20–0.75) §	5.85 (2.89–11.71) §	5.88 (2.99–11.50) §	0.15 (0.07–0.30) §	1.54 (1.14–2.09) §	0.73 (0.34–1.57)

Odd ratios and 95% confidence intervals are shown in the table.

§: significant P < 0.05; GDM: gestational diabetes mellitus; PIH: pregnancy-induced hypertension; LGA: large for gestational age; SGA: small for gestational age; P-CS: primary caesarean section.

We divided the study population into three groups based on pre-pregnancy BMI: Underweight (n = 450; BMI < 18.5 kg/m^2^), Normal (n = 2,067; 18.5 ≤ BMI < 25.0 kg/m^2^), and Obese (n = 262; BMI ≥ 25.0 kg/m^2^). Table 4 shows how different the ORs of developing RGWG-related adverse pregnancy outcomes in these three BMI groups are. In the Normal group, RGWG at early pregnancy was significantly associated with elevated risks of GDM (OR = 2.00), PIH (OR = 2.34), LGA infants (OR = 2.67), macrosomia (OR = 3.02), and P-CS (OR = 2.08), similar to the entire study population (Table 3). Similarly, RGWG at early pregnancy in the Normal group was associated with a low risk of SGA (OR = 0.47), as in the entire study population (OR = 0.67). However, in the Underweight group, early RGWG was not significantly associated with any of the adverse pregnancy outcomes. In the Obese group, early RGWG was only associated with a high risk of PIH (OR = 4.41), whereas late RGWG was significantly associated with a high risk of macrosomia (OR = 2.72) and a low risk of preterm (OR = 0.42) and SGA (OR = 0.39).

**Table 4:** Risk analysis of developing adverse pregnancy outcomes–pre-pregnancy BMI.

		GDM	PIH	Preterm	Macrosomia	LGA	SGA	P_CS	Low APGAR
**Underweight**	Early pregnancy	1.20 (0.36–4.01)	1.66 (0.04–64.50)	0.58 (0.05–6.35)	4.76 (0.79–28.68)	4.76 (0.79–28.68)	0.51 (0.11–2.27)	1.59 (0.69–3.67)	1.91 (0.22–16.44)
	Mid pregnancy	0.69 (0.29–1.66)	2.56 (0.39–16.75)	1.47 (0.38–5.72)	1.18 (0.17–8.11)	1.18 (0.17–8.11)	0.60 (0.23–1.53)	2.87 (1.44–5.69) §	0.68 (0.08–5.46)
	Late pregnancy	0.19 (0.07–0.55) §	4.44 (0.71–27.97)	0.99 (0.19–5.12)	0.53 (0.04–6.26)	0.98 (0.08–12.58)	1.28 (0.47–3.52)	0.70 (0.37–1.35)	0.40 (0.06–2.79)
	Early and mid pregnancy	0.92 (0.20–4.35)	7.90 (0.55–114.22)	2.73 (0.32–23.34)	7.15 (0.45–114.58)	7.41 (0.59–92.67)	0.23 (0.04–1.38)	6.40 (1.98–20.70) §	3.61 (0.27–48.68)
	Mid and late pregnancy	0.09 (0.02–0.42) §	48.29 (0.77–3031.70)	0.57 (0.06–5.59)	0.50 (0.01–17.26)	0.50 (0.01–17.26)	0.21 (0.05–0.97) §	2.07 (0.80–5.37)	0.03 (0.00–0.60) §
	Whole pregnancy	0.10 (0.01–0.77) §	34.11 (1.28–908.15) §	0.39 (0.05–3.31)	3.61 (0.14–91.40)	5.26 (0.29–96.06)	0.16 (0.03–0.77) §	2.46 (1.03–5.85) §	0.23 (0.02–3.56)
**Normal**	Early pregnancy	2.00 (1.26–3.16) §	2.34 (1.11–4.93) §	0.90 (0.47–1.74)	3.20 (1.52–6.71) §	2.67 (1.32–5.43) §	0.47 (0.24–0.92) §	2.08 (1.50–2.90) §	1.51 (0.63–3.61)
	Mid pregnancy	0.95 (0.67–1.33)	1.35 (0.79–2.29)	1.40 (0.85–2.29)	1.50 (0.89–2.54)	1.48 (0.89–2.46)	0.77 (0.44–1.35)	1.06 (0.83–1.37)	1.11 (0.58–2.10)
	Late pregnancy	0.34 (0.24–0.48) §	1.26 (0.82–1.94)	0.52 (0.33–0.82) §	1.37 (0.92–2.02)	1.43 (0.98–2.09)	1.00 (0.60–1.67)	0.73 (0.58–0.92) §	0.82 (0.48–1.43)
	Early and mid pregnancy	2.63 (1.56–4.44) §	3.87 (1.65–9.10) §	0.83 (0.38–1.83)	4.70 (2.08–10.61) §	3.74 (1.66–8.44) §	0.29 (0.11–0.80) §	3.85 (2.49–5.98) §	1.36 (0.55–3.34)
	Mid and late pregnancy	0.25 (0.15–0.43) §	1.86 (0.85–4.05)	1.36 (0.65–2.85)	2.94 (1.41–6.12) §	3.14 (1.56–6.31) §	0.78 (0.34–1.78)	0.72 (0.50–1.03)	1.13 (0.43–2.96)
	Whole pregnancy	0.69 (0.37–1.28)	4.57 (1.63–12.82) §	0.35 (0.15–0.82) §	8.81 (3.85–20.17) §	9.11 (4.02–20.61) §	0.13 (0.05–0.32) §	1.67 (1.16–2.39) §	1.01 (0.40–2.55)
**Obese**	Early pregnancy	1.65 (0.80–3.41)	4.41 (1.28–15.21) §	1.13 (0.34–3.73)	0.78 (0.34–1.74)	0.88 (0.41–1.90)	2.32 (0.41–13.24)	0.81 (0.44–1.50)	0.53 (0.19–1.46)
	Mid pregnancy	1.09 (0.62–1.91)	0.78 (0.37–1.66)	0.78 (0.30–2.05)	1.68 (0.66–4.26)	1.62 (0.70–3.72)	0.84 (0.28–2.53)	0.86 (0.49–1.49)	3.56 (0.21–59.25)
	Late pregnancy	0.19 (0.09–0.43) §	0.71 (0.34–1.51)	0.42 (0.18–0.98) §	2.72 (1.04–7.12) §	2.32 (0.99–5.42)	0.39 (0.15–0.98) §	0.84 (0.48–1.47)	0.48 (0.18–1.27)
	Early and mid pregnancy	2.09 (0.71–6.13)	5.42 (0.95–31.11)	0.68 (0.12–3.86)	0.77 (0.20–3.02)	1.10 (0.31–3.89)	1.61 (0.20–13.29)	0.87 (0.34–2.22)	0.32 (0.04–2.61)
	Mid and late pregnancy	0.18 (0.07–0.48) §	0.42 (0.12–1.48)	1.21 (0.28–5.18)	3.43 (0.93–12.67)	2.96 (0.91–9.58)	0.28 (0.05–1.47)	0.70 (0.32–1.56)	1.28 (0.16–9.98)
	Whole pregnancy	0.56 (0.20–1.56)	2.34 (0.46–11.83)	0.43 (0.11–1.75)	2.95 (0.75–11.61)	3.36 (0.96–11.76)	0.52 (0.08–3.38)	0.84 (0.37–1.88)	0.26 (0.05–1.35)

Odd ratios and 95% confidence intervals are shown in the table.

§: significant P < 0.05; GDM: gestational diabetes mellitus; PIH: pregnancy-induced hypertension; LGA: large for gestational age; SGA: small for gestational age; P-CS: primary caesarean section.

## Discussion

In this study, we showed that early RGWG is highly associated with developing adverse pregnancy outcomes, suggesting that control of early weight gain is important in preventing such pregnancy-related adverse outcomes. To the best of our knowledge, this is the first study to clarify the relationship between gestational stage-specific weight gain and adverse outcomes in a large cohort.

In 2009, the Institute of Medicine (IOM) updated its guidelines for total and rate of weight gain during pregnancy based on pre-pregnancy BMI category. The recommendation suggested further studies should be performed on the effects of RGWG at different stages of gestation rather than total weight gain. In the current study, we examined the relationship between RGWG at early, mid, and late pregnancy and adverse pregnancy outcomes and have demonstrated that RGWG at early and late pregnancy, but not mid pregnancy, is significantly associated with developing GDM, PIH, LGA infants, macrosomia, and P-CS.

Others studies have shown that GWG before OGTT is associated with increasing risk of impaired glucose tolerance [11] or GDM development [10]. GWG in the first and second trimesters, but not third trimester, was shown to be predictive of newborn weight [13]. Early pregnancy BMI had a significant effect on birth weight, although the degree of the effect was different between non-Europeans and Europeans [7].

However, the results of our study demonstrate that RGWG in early pregnancy predicts adverse pregnancy outcomes, but RGWG in mid pregnancy is not significantly associated with any adverse pregnancy outcomes. These findings suggest that the significant effects of RGWG, until OGCT (early and mid pregnancy), on adverse pregnancy outcomes are largely attributable to the independent effect of weight changes in early pregnancy, but not mid pregnancy [14–16].

Our results indicate that the relationship between RGWG and adverse pregnancy outcomes is dramatically different among the three pre-pregnancy BMI groups (Underweight, Normal, and Obese). At early pregnancy, no significant relationship between RGWG and any adverse pregnancy outcomes was identified in the Underweight group. In the Normal group, however, RGWG was significantly associated with GDM, PIH, LGA infants, macrosomia, P-CS, and SGA infants, whereas early RGWG was only associated with a high risk of PIH in the Obese group. These results suggest that the recommended RGWG or GWG should be adjusted based on pre-pregnancy BMI and pregnancy term (early, mid, and late pregnancy).

There are few previous reports on the importance of early GWG on pregnancy outcomes [9, 10, 17]. Excessive early weight gain over the 2009 IOM recommendation [9] and first-trimester RGWG [10] were shown to be associated with the development of GDM. Another study have shown that GWG in early pregnancy (up to 18 weeks of gestation) is a risk factor for gestational hypertension and preeclampsia [17]. However, these previous studies were limited to a specific period, such as early pregnancy and a few adverse pregnancy outcomes, such as GDM or PIH. Our study, on the contrary, examined a comprehensive list of outcomes, including P-CS, low APGAR score, and preterm birth, in examining their relationship to RGWGs in early, mid, and late pregnancy. Moreover, our study demonstrates that the effects of RGWGs on the development of various adverse pregnancy outcomes are dependent on pre-pregnancy BMI.

Earlier diagnosis of adverse pregnancy complications such as GDM is a major topic for clinicians. Currently-available standard OGCT/OGTT for GDM diagnosis is done at 24–28 weeks of gestation and is considered to be too late for prevention of diabetes complications. Some other non-invasive measures, such as detection of placental miRNAs [18] or early RGWG, might be of additive and predictive value for reducing the frequency and severity of adverse pregnancy outcomes. Our study shows that RGWGs up to OGCT or during the entire pregnancy have higher odds of developing GDM and P-CS, but RGWG in late pregnancy has lower odds of GDM and P-CS. Therefore, avoiding excessive RGWG during early pregnancy may be an effective way to prevent adverse outcomes, and doctors should take preemptive action with pregnant women so that they do not gain an excessive amount of weight, even at early pregnancy.

However, the reason for this inverse discrepancy between specific pregnancy period and GDM risk is not clear. One possible reason is that the women diagnosed with GDM may have undergone a strict weight control in late pregnancy. Different types of weight gain may play a role since early GWG is related to maternal body fat followed by an increase in insulin resistance, whereas later GWG is closely related to fat-free mass [19].

In the clinical setting, it will be very helpful if there are serum markers that are strongly associated with GWG to predict adverse pregnancy outcomes. It has not been well established which serum markers or cytokines are related to early RGWG and adverse pregnancy outcomes. However, it was demonstrated that maternal plasma adiponectin concentrations in early pregnancy were statistically significantly lower in women who developed GDM, as compared with controls and maternal plasma adiponectin levels that were inversely correlated with BMI in early pregnancy among GDM patients [20]. Another study showed that serum maternal leptin levels at 6–8 weeks of pregnancy were significantly correlated with BMI, but the correlation between serum leptin levels and BMI was weakened with increased gestational age [21]. Clearly, more studies are needed to further elucidate the early biomarkers or cytokines such as adiponectin, leptin, estriol, or their mechanisms which may play a part in regulating GWG or pregnancy outcomes.

Recent studies showed that gestational weight gain varies by pre-pregnancy obesity classification and obese women gain more excessive GWG than recommended by IOM guidelines and are at high risk for LGA infants [22, 23]. The pattern of GWG was also found to be significantly associated with abnormal fetal growth [24]. Our data, as well as other researchers’, suggest that the current IOM recommendation may be stratified further by timing of GWG or severity of obesity in pre-pregnancy or pattern of GWG.

The limitation of this study is that the study cohort is from a single university hospital, thus may not represent the entire population. No information regarding the diet and physical activities during pregnancy was available in the medical records; moreover, it was not possible to correlate them with the different patterns of ORs in late pregnancy. Besides, no biochemical and biophysical factors were examined for potential association with early weight gain, therefore, we are currently investigating these related factors.

To fully understand the impact of gestational weight gain for women and their offspring, we need consistent definitions of GWG, including weight gain above IOM guidelines during pregnancy, better assessment of confounders in their analyses, and conduct studies with longer follow-up of outcomes.

## Conclusions

RGWG of early pregnancy is significantly associated with multiple adverse effects on pregnancy outcomes, therefore, it is critical to properly manage maternal pregnancy weight, even during early pregnancy, to reduce the risk of developing adverse pregnancy outcomes.

## Supporting Information

S1 File Chart data. (XLSX)
